# An insert-based enzymatic cell culture system to rapidly and reversibly induce hypoxia: investigations of hypoxia-induced cell damage, protein expression and phosphorylation in neuronal IMR-32 cells

**DOI:** 10.1242/dmm.013078

**Published:** 2013-09-05

**Authors:** Ying Huang, Karina Zitta, Berthold Bein, Markus Steinfath, Martin Albrecht

**Affiliations:** 1 Department of Anaesthesiology and Intensive Care Medicine, University Hospital Schleswig-Holstein Schwanenweg 21, 24105 Kiel, Germany

## Abstract

Ischemia-reperfusion injury and tissue hypoxia are of high clinical relevance because they are associated with various pathophysiological conditions such as myocardial infarction and stroke. Nevertheless, the underlying mechanisms causing cell damage are still not fully understood, which is at least partially due to the lack of cell culture systems for the induction of rapid and transient hypoxic conditions. The aim of the study was to establish a model that is suitable for the investigation of cellular and molecular effects associated with transient and long-term hypoxia and to gain insights into hypoxia-mediated mechanisms employing a neuronal culture system. A semipermeable membrane insert system in combination with the hypoxia-inducing enzymes glucose oxidase and catalase was employed to rapidly and reversibly generate hypoxic conditions in the culture medium. Hydrogen peroxide assays, glucose measurements and western blotting were performed to validate the system and to evaluate the effects of the generated hypoxia on neuronal IMR-32 cells. Using the insert-based two-enzyme model, hypoxic conditions were rapidly induced in the culture medium. Glucose concentrations gradually decreased, whereas levels of hydrogen peroxide were not altered. Moreover, a rapid and reversible (onoff) generation of hypoxia could be performed by the addition and subsequent removal of the enzyme-containing inserts. Employing neuronal IMR-32 cells, we showed that 3 hours of hypoxia led to morphological signs of cellular damage and significantly increased levels of lactate dehydrogenase (a biochemical marker of cell damage). Hypoxic conditions also increased the amounts of cellular procaspase-3 and catalase as well as phosphorylation of the pro-survival kinase Akt, but not Erk1/2 or STAT5. In summary, we present a novel framework for investigating hypoxia-mediated mechanisms at the cellular level. We claim that the model, the first of its kind, enables researchers to rapidly and reversibly induce hypoxic conditions *in vitro* without unwanted interference of the hypoxia-inducing agent on the cultured cells. The system could help to further unravel hypoxia-associated mechanisms that are clinically relevant in various tissues and organs.

## INTRODUCTION

Ischemia-reperfusion injury and tissue hypoxia are of high clinical relevance. Besides the occurrence of perioperative ischemia and hypoxia in various organs and tissues, myocardial infarction and stroke are characterized by a rapid decrease in tissue oxygenation, which in turn induces molecular events that lead to cell death, tissue damage and inflammation (Eltzschig and Eckle, 2011; Krohn et al., 2008). An understanding of the hypoxia-associated cellular and molecular mechanisms is essential for the development of new and effective strategies to reduce ischemia-reperfusion injury and hypoxia-mediated cell damage, leading to an improved clinical outcome and reduced mortality.

Different *in vitro* models (e.g. hypoxic chambers, chemical or enzymatic generation of hypoxia) have been employed in the past to mimic the clinical scenario of tissue hypoxia and to unravel the underlying mechanisms (Askoxylakis et al., 2011; Lièvre et al., 2000; Saxena et al., 2012; Yu et al., 2007). Unfortunately, all of the models established so far have major drawbacks. Either they are not suitable for the clinically relevant rapid induction and/or termination of hypoxia (hypoxic chambers) or it is not possible to exclude potential side effects that are caused by the direct addition of hypoxia-inducing chemicals or enzymes to the culture medium and therefore to the cells within, which might impair the transferability of the results to the *in vivo* situation.

To overcome these problems, we have for the first time established a simple and easy-to-handle insert-based enzymatic cell culture system for the rapid and reversible induction of hypoxia in which the cells do not come into contact with the hypoxia-inducing agents. Our results obtained with neuronal cells show that the system can be used to mimic the major events of tissue hypoxia and might therefore facilitate the search for strategies to reduce ischemia-reperfusion injury.

## RESULTS

### Setup of the enzyme-based insert system

**Fig. 1.**
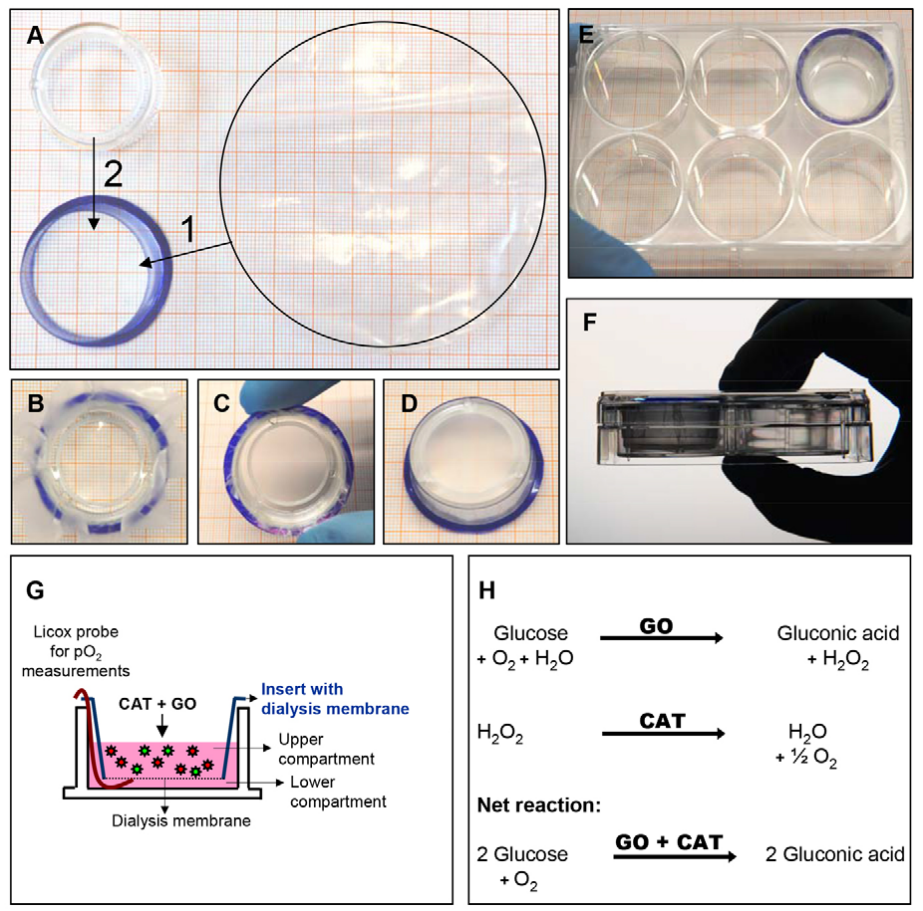
**Assembly and functionality of the insert-based two-enzyme hypoxia system.** (A–F) Commercially available six-well inserts from which the bottom membrane was removed are used as a framework for the assembly of a semipermeable dialysis membrane. (A–D) The basic steps of insert assembly. 1 and 2 in A describe the order in which the steps are performed: 1, insert the semipermeable membrane; 2, insert the plastic ring. (E,F) The assembled insert in a six-well plate. (G) Addition of glucose oxidase and catalase into the upper compartment containing standard cell culture medium leads to the generation of hypoxia in the lower compartment. (H) Schematic depiction of the glucose-oxidase- and catalase-dependent reactions that lead to hypoxic culture conditions. CAT, catalase; GO, glucose oxidase.

Induction of hypoxic conditions was performed by employing an enzymatic model consisting of glucose oxidase and catalase in combination with a standard six-well system (for details see Materials and Methods). To avoid contact of the hypoxia-inducing enzymes with the cells, membrane-denuded cell culture inserts were used as a framework on which a dialysis membrane with 10- to 20-kDa cutoff was assembled (Fig. 1A–F). Replacing the semipermeable membrane by the dialysis membrane results in a restriction of the hypoxia-inducing enzymes to the insert system, while oxygen is deployed from the culture medium of the lower compartment containing the cells (Fig. 1G,H).

**RESOURCE IMPACT**

**Background**

Tissue hypoxia and ischemia-reperfusion injury are common perioperative complications. In addition, pathophysiological conditions such as circulatory failure, myocardial infarction and stroke are characterized by a rapid decrease in tissue oxygenation, which induces molecular events that lead to cell death, tissue damage and inflammation. A better understanding of the cellular and molecular mechanisms associated with hypoxia is essential for the development of new and effective clinical strategies to reduce ischemiareperfusion-mediated cell damage. However, all the *in vitro* models that have been used in the past for the investigation of the cellular and molecular events that are associated with hypoxia have major drawbacks. In most models, it is not possible to rapidly induce and terminate hypoxia and, in those in which hypoxia is generated through the direct addition of hypoxia-inducing agents to the cell culture medium, unwanted side effects can impair the transferability of the results to the *in vivo* situation.

**Results**

In this study, the authors develop an easy-to-handle, semipermeable-membrane-based insert system that works together with the hypoxia-inducing enzymes glucose oxidase and catalase to rapidly and reversibly generate hypoxic conditions in culture without the cells coming into contact with the hypoxia-inducing agents. The authors show first that hypoxia is rapidly and reversibly induced. Then, using a neuronal cell line, they demonstrate that characteristic hypoxia-mediated cell responses (including cellular damage, and protein expression and phosphorylation) are induced in their cell culture system.

**Implications and future directions**

The new experimental approach described in this study replicates the fast-onset hypoxia that is commonly observed in circulatory failure, myocardial infarction and stroke. It provides a new and valuable tool that could help to unravel clinically relevant hypoxia-associated mechanisms and to promote a better understanding of ischemic conditioning as well as of tissue hypoxia and ischemia-reperfusion injury. The *in vitro* model presented here could also facilitate the search for strategies to prevent and/or reduce the tissue and organ damage caused by hypoxia and ischemia-reperfusion injury.

### Hypoxic conditions are rapidly and reversibly induced by employing the insert-based enzymatic system

**Fig. 2.**
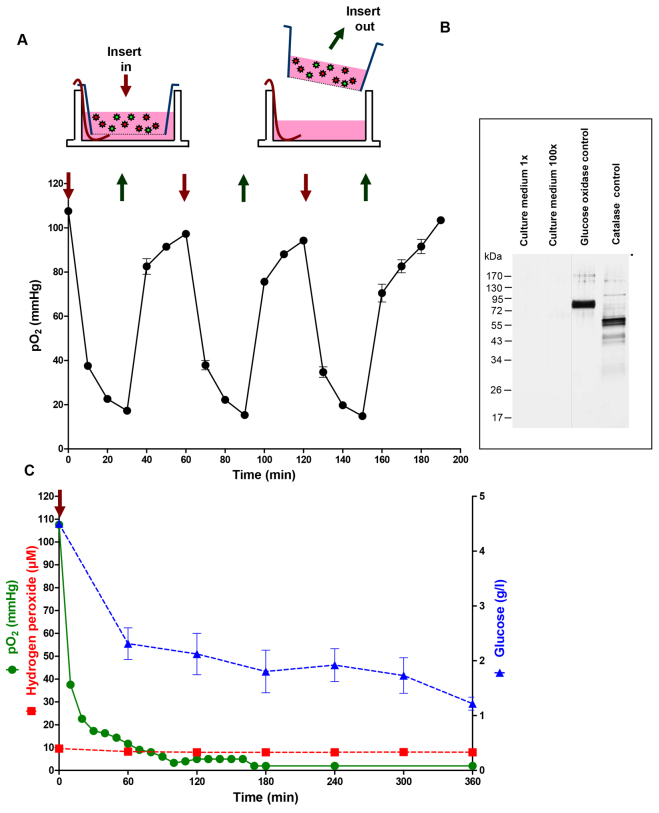
**Characterization of transient and long-term hypoxic conditions induced by the insert-based two-enzyme system.** (A) Application of the enzyme-containing insert (red arrow) results in a rapid decrease in pO_2_. After removing the insert (dark green arrow), pO_2_ quickly increases to almost baseline levels. This on-off procedure can be repeated, yielding similar pO_2_ profiles each time. (B) Silver staining experiments show that the hypoxia-inducing enzymes are restrained within the insert and do not pass the semipermeable membrane. (C) Applying the enzyme-containing insert for a prolonged time period results in a decrease of pO_2_ to values as low as 2.00 mmHg. Hypoxic conditions are stable for at least 6 hours. During the experiment, glucose concentrations gradually decrease, whereas hydrogen peroxide levels keep at a steady state. Bars denote mean ± s.e.m. of three experiments.

To evaluate the temporal kinetics of hypoxia induction and to check whether the insert-based model was suitable for the rapid and reversible (on-off) generation of hypoxia, repeated cycles of hypoxia induction and reoxygenation were performed by the addition and subsequent removal of inserts containing glucose oxidase and catalase. Adding the enzyme-containing insert (at time 0) resulted in a rapid decrease in partial pressure of oxygen (pO_2_), whereas, after removing the insert 30 minutes later, pO_2_ quickly recovered to almost baseline levels (pO_2_ 0 minutes: 107.60±0.99 mmHg; pO_2_ 30 minutes: 17.30±0.51 mmHg; pO_2_ 60 minutes: 97.23±0.20 mmHg). This procedure was repeated twice, yielding similar results concerning the time profile of hypoxia and reoxygenation (Fig. 2A).

Silver stainings were performed with native and 100-times concentrated culture media of the lower compartment, showing that neither of the hypoxia-inducing enzymes (glucose oxidase, catalase) penetrated the semipermeable membrane of the insert during the experiment (Fig. 2B).

Additional long-term hypoxia studies were carried out by applying the enzyme containing inserts continuously for up to 6 hours (Fig. 2C). The results showed that, after 70 minutes, pO_2_ reached levels below 10 mmHg (pO_2_ 70 minutes: 9.00±0.58 mmHg). This value further decreased to 5 mmHg after 120 minutes (pO_2_ 120 minutes: 5.00±0.00 mmHg) and 2 mmHg after 170 and 360 minutes (pO_2_ 170/360 minutes: 2.00±0.00 mmHg). Because the enzymatic reaction leading to hypoxia is dependent on the glucose concentration in the culture medium (Fig. 1H), we evaluated the concentrations of glucose at different time points, showing a reduction from the initial 4.5 g/l ([Glc] 0 minutes: 4.50±0.02 g/l) to 1.2 g/l after 6 hours of hypoxia ([Glc] 360 minutes: 1.22±0.07 g/l). Concentrations of hydrogen peroxide were kept at steady-state levels of under 10 μM throughout the experiment ([H_2_O_2_] 0 minutes: 9.57±0.00 μM; [H_2_O_2_] 360 minutes: 7.96±0.67 μM; Fig. 2C).

### Hypoxic conditions generated by using the insert-based enzymatic system result in damage of cultured neuronal cells

**Fig. 3.**
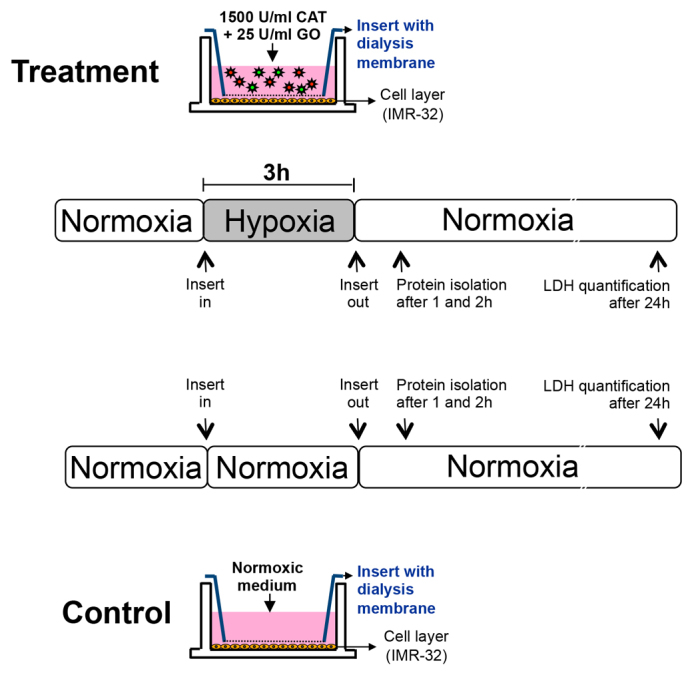
**Experimental setting and time frame of the study.** CAT, catalase; GO, glucose oxidase.

**Fig. 4.**
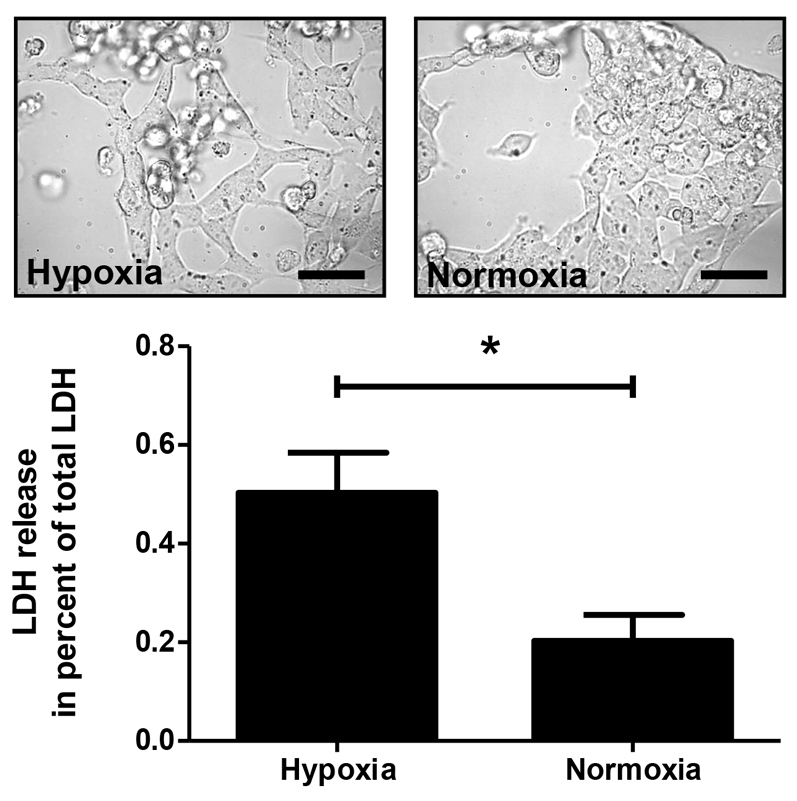
**Hypoxia generated by using the insert-based enzymatic system damages cultured neuronal cells.** Cells subjected to hypoxia show morphological changes such as cell rounding and detachment from the growth surface. Scale bars: ∼30 μm. LDH measurements in the culture media confirm these results and reveal an increase of LDH in the hypoxia group. Bars denote mean ± s.e.m. of five experiments; *P<0.05.

To evaluate the effects of hypoxia on cell damage and morphology, IMR-32 cells were exposed to a total of 3 hours of hypoxia. Morphological analyses and measurements of lactate dehydrogenase (LDH) as a marker for cell damage were performed 24 hours after the end of hypoxia (Fig. 3). Compared with the normoxia control, cells subjected to hypoxia showed distinct morphological changes such as cell rounding and detachment from the growth surface. LDH was measured in culture supernatants and was relativized to the levels of total LDH obtained after cell lysis. The results revealed a 2.5-fold increase of LDH in the hypoxia group compared with the normoxia control (hypoxia: 0.50±0.08 a.u.; normoxia: 0.20±0.05 a.u.; *P*<0.05; Fig. 4).

### Hypoxic conditions generated with the insert-based enzymatic system regulate protein expression and phosphorylation

**Fig. 5.**
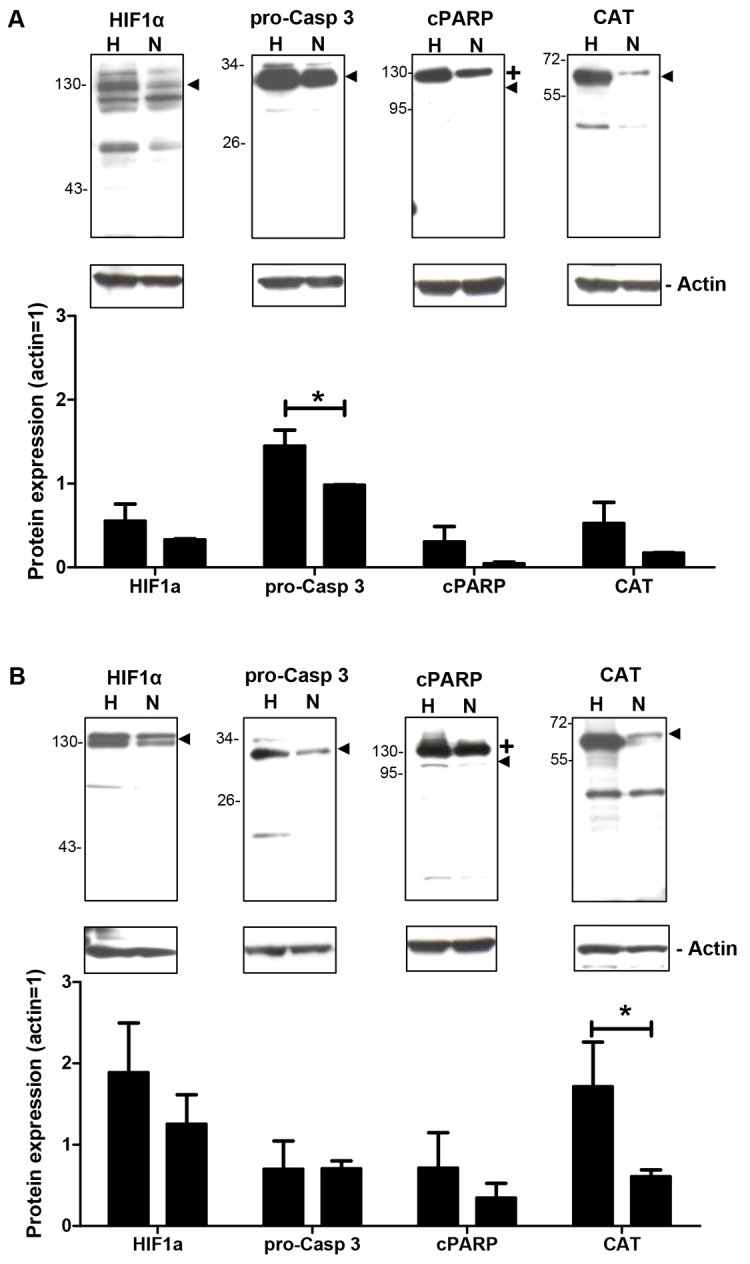
**Effects of hypoxia generated by using the insert-based enzymatic system on the expression of hypoxia-associated proteins.** Several hypoxia-associated proteins are differentially expressed between the hypoxia (H) and normoxia (N) groups at 1 hour (A) and 2 hours (B) after the hypoxic insult. One representative western blotting experiment is shown above the columns. Arrowheads denote the specific protein band detected by the respective antibody. Bars denote mean ± s.e.m. of three experiments; *P<0.05; +, uncleaved PARP.

**Fig. 6.**
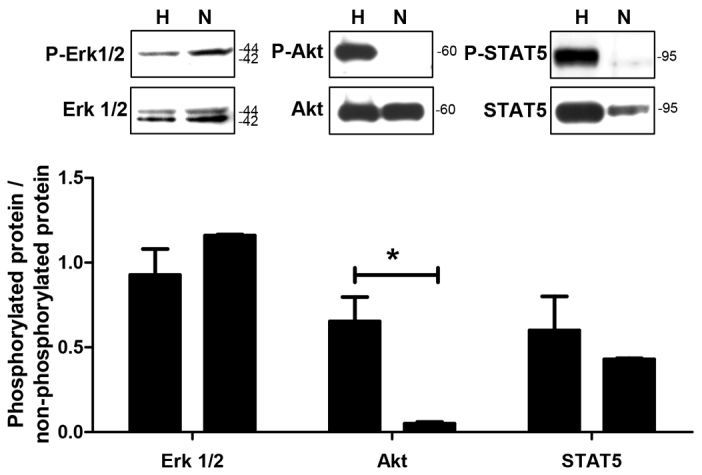
**Effects of hypoxia generated by using the insert-based enzymatic system on the phosphorylation of cell signalling molecules.** Several molecules associated with cellular signalling and survival events are differentially phosphorylated between the hypoxia (H) and normoxia (N) groups at 2 hours after the hypoxic insult. One representative western blotting experiment is shown above the columns. Bars denote mean ± s.e.m. of three experiments; *P<0.05.

In almost all cell types and tissues, hypoxia is associated with profound changes in gene and protein expression (Bruick, 2003; Chi et al., 2006). To investigate whether hypoxic conditions generated using the insert-based enzymatic system lead to alterations in the expression and phosphorylation of typical hypoxia-associated proteins, western blotting experiments were performed with antibodies directed against the following molecules: hypoxia inducible factor-1alpha (HIF-1α), procaspase-3, cleaved poly (ADP-ribose) polymerase (cPARP), catalase (CAT), phosphorylated and total extracellular signal-regulated kinase 1/2 (P-Erk1/2 and Erk1/2), phosphorylated and total protein kinase B (P-Akt and Akt), and phosphorylated and total signal transducer and activator of transcription 5 (P-STAT5 and STAT5). For all molecules, changes in the protein expression and/or phosphorylation were detected under hypoxia. However, statistically significant differences, namely an increased protein expression or phosphorylation, were only observed for procaspase-3 at 1 hour after hypoxia (hypoxia: 1.45±0.19 a.u.; normoxia: 0.98±0.01 a.u.; *P*<0.05; Fig. 5A), for catalase at 2 hours after hypoxia (hypoxia: 1.71±0.55 a.u.; normoxia: 0.61±0.09 a.u.; *P*<0.05; Fig. 5B) and for P-Akt at 2 hours after hypoxia (hypoxia: 0.65±0.14 a.u.; normoxia: 0.05±0.01 a.u.; *P*<0.05; Fig. 6).

## DISCUSSION

Tissue hypoxia is frequently found under various pathophysiological conditions, such as circulatory failure, myocardial infarction and cerebral ischemia (Garin et al., 2005; Li and Jackson, 2002; McCord, 1985; Michiels, 2004). Owing to the high incidence and clinical relevance of tissue hypoxia and ischemia-reperfusion injury, an understanding of the hypoxia-associated cellular and molecular mechanisms is essential for the development of new and effective strategies to reduce ischemia-reperfusion- and tissue-hypoxia-mediated cell damage.

An elegant and straightforward method for the investigation of hypoxia-associated mechanisms is the use of cell culture systems. So far, different *in vitro* models (e.g. hypoxic chambers, chemical or enzymatic generation of hypoxia) have been employed to induce hypoxic conditions in cultures of cell lines and primary cells and to evaluate the effects as well as underlying mechanisms of *in vitro* hypoxia. Unfortunately, all of the currently described models have major drawbacks. Various studies have employed so-called hypoxic chambers, in which the cell cultures are exposed to an anoxic or hypoxic atmosphere in a closed chamber (Alqawi et al., 2007; Garayoa et al., 2003; Li et al., 2003). Using hypoxic chambers, the gaseous mixture can in fact be precisely adjusted, but a rapid induction and termination of hypoxic conditions within the cell culture medium cannot be achieved, which is due to the slow equilibration of pO_2_ between the gaseous and the aqueous phase of the system. Several authors have shown that, in hypoxic chambers, between 3 and 24 hours are required for the oxygen concentrations in the liquid phase to reach equilibrium with the gas mixture in the chamber (Allen et al., 2001; Mueller et al., 2009), making the clinically relevant rapid induction of hypoxic conditions impossible in these models. Hypoxic conditions can also be induced by the addition of cobalt chloride (Cheng et al., 2013; Garayoa et al., 2000; Gotoh et al., 2012) or sodium dithionite (Abudara et al., 2002; Mancarella et al., 2011; Yu et al., 2007), or by the application of the hypoxia-inducing enzymes glucose oxidase and catalase (Askoxylakis et al., 2011; Baumann et al., 2008; Mueller et al., 2009). Although the last-mentioned systems are easy to handle and rapidly induce hypoxic conditions, one of their major disadvantages is that the cells are in direct contact with the hypoxia-inducing agents. Therefore, the cells’ behaviour and response might be altered and might not reflect the *in vivo* situation anymore.

To overcome the deficiencies of currently available hypoxia models, we have established an insert-based enzymatic cell culture system for the rapid and reversible induction of hypoxia in which the cells do not come into contact with the hypoxia-inducing agents. Employing the two enzymes glucose oxidase and catalase, as well as glucose-containing culture medium and a culture insert containing a dialysis membrane with a 10- to 20-kDa cutoff, hypoxic conditions (pO_2_ ≤10 mmHg) were achieved after 70 minutes. Hypoxia could be terminated and reinduced by simply removing and reapplying the enzyme-containing insert at any desired time point. Concerning the induction of prolonged hypoxic conditions, we showed that, in our model, hypoxia could be maintained for at least 6 hours. Concentrations of glucose in the respective culture medium decreased from the initial 4.5 g/l to 1.2 g/l after 6 hours, and hydrogen peroxide concentrations remained at steady-state levels of under 10 μM throughout the experiment. Although, in our insert-based system, induction of hypoxia is somewhat slower compared with the direct addition of the enzymes (Zitta et al., 2010b), reduction of glucose concentrations and hydrogen peroxide levels are in the range of what has been reported by others working with enzyme-driven hypoxia systems (Baumann et al., 2008; Mueller et al., 2009). It should also be mentioned that the reduction of glucose concentrations throughout the experiment and the increased levels of hydrogen peroxide (compared with normoxic conditions) ideally reflect the *in vivo* situation of hypoxia and ischemia-reperfusion injury, in which glucose depletion and accumulation of reactive oxygen species occur and are believed to play a central role in cell death and tissue damage (Baudry et al., 2008; Gawlitta et al., 2007; Goldberg and Choi, 1993; Guo et al., 2011).

Because cerebral ischemia and brain hypoxia are clinically highly relevant conditions (Robertson and Perlman, 2006), we employed the neuronal cell line IMR-32 (Tumilowicz et al., 1970) to validate the suitability of the established system for the induction of a hypoxia-mediated cell response and investigation of the associated mechanisms. A total of 3 hours of hypoxia resulted in clear morphological signs of cell damage as well as a 2.5-fold increase in LDH levels. These results are consistent with data from a previous study, in which we showed that 2 hours of direct enzyme-induced hypoxia increased cell damage in IMR-32 cultures ∼4.5-fold compared with normoxic conditions (Zitta et al., 2010a). The somewhat delayed and less distinct cell damage in the insert-based system could be explained by the fact that the generation of hypoxia is slower in the latter model, giving the cells more time to respond with protective mechanisms such as an increased expression of antioxidant catalase or phosphorylation of pro-survival Akt (see below).

To further evaluate the insert-based hypoxia model, western blotting experiments for typical hypoxia-associated molecules involved in cell death [procaspase-3 (Delivoria-Papadopoulos et al., 2008; Garnier et al., 2004), cPARP (Araya et al., 1998; Hong et al., 2004)], antioxidant defence [catalase (Guo et al., 2011; Vergara et al., 2012)] and hypoxia mediated gene regulation [HIF-1α (Greijer et al., 2005; Semenza, 2012)] were performed. A statistically significant increase in protein expression after hypoxia was only detected for catalase and procaspase-3, whereas HIF-1α and cPARP protein levels were by trend augmented under hypoxic conditions. The results obtained for HIF-1α and catalase confirm our previous studies in which we also employed IMR-32 cells and showed that 2 hours of hypoxia increased the protein expression and/or stability of catalase (Huang et al., 2013) and HIF-1α (Zitta et al., 2010a). PARP is a 116-kDa nuclear poly (ADP-ribose) polymerase involved in DNA repair in response to environmental stress (Satoh and Lindahl, 1992). PARP helps cells to maintain viability, and cleavage of PARP, which *in vivo* is mainly accomplished by caspase-3 (Cohen, 1997), serves as a marker of cells undergoing apoptosis (Oliver et al., 1998). Our results show increased PARP cleavage under hypoxic conditions, suggesting that hypoxia induces apoptotic events in IMR-32 cells. Interestingly, our western blotting experiments do not reveal increased procaspase-3 cleavage into active caspase-3. This observation might indicate that, under *in vitro* conditions, other caspases apart from caspase-3 might also be able to cleave PARP. Concerning the phosphorylation of the key signalling molecules Erk1/2, Akt and STAT5 (Balmanno and Cook, 2009; Jiang and Liu, 2008; Schindler, 2002), we found a statistically significant increase of Akt phosphorylation in IMR-32 cells 2 hours after hypoxia, whereas Erk1/2 and STAT5 were not regulated. Phosphorylated Akt can promote cell survival via phosphorylating BAD, a member of the Bcl-2 family (Datta et al., 1997; del Peso et al., 1997), and might also activate NF-κB via regulating IκB kinase leading to the transcription of pro-survival genes (Barkett and Gilmore, 1999; Faissner et al., 2006; Lauder et al., 2001). Therefore, the increased phosphorylation of Akt in IMR-32 cells might point towards protective mechanisms counteracting the potentially apoptotic events induced by hypoxia. Taken together, the biochemical results obtained with IMR-32 cells underline the functionality of the insert-based hypoxia model for the investigation of cellular and molecular mechanisms associated with hypoxic and ischemic conditions.

In summary, the model presented is the first of its kind and we claim that this approach is particularly advantageous for researchers in two major respects: (1) the system facilitates the rapid and reversible induction of hypoxic conditions *in vitro* without direct contact between the hypoxia-inducing agents and cultured cells, thereby reducing unwanted side effects; and (2) our model could help to further unravel hypoxia-associated mechanisms that are clinically relevant in various tissues and organs, and might facilitate the understanding of ischemia-reperfusion injury as well as ischemic conditioning.

## MATERIALS AND METHODS

### Setup of the enzyme-based insert system

Induction of hypoxic conditions was performed by employing an enzymatic system (Baumann et al., 2008; Huang et al., 2013; Mueller et al., 2009; Zitta et al., 2012a; Zitta et al., 2012b; Zitta et al., 2010a; Zitta et al., 2010b) consisting of glucose oxidase (Sigma-Aldrich, Schnelldorf, Germany; final concentration 25 U/ml) and catalase (Sigma-Aldrich, Schnelldorf, Germany; final concentration 1500 U/ml) in DMEM high-glucose medium with 1% FCS (PAA, Coelbe, Germany) in combination with a standard six-well system (NUNC, Roskilde, Denmark). To avoid contact of the hypoxia-inducing enzymes with the cells, membrane-denuded cell culture inserts (NUNC, Roskilde, Denmark) were used as a framework on which a dialysis membrane (cutoff 10–20 kDa; Nadir-dialysis tubing, Duren, Germany) was assembled (Fig. 1A–F). Note that conventional cell culture insert systems with a semipermeable membrane are not suitable because their pore size (≥0.4 μm) allows the free passage of glucose oxidase and catalase molecules. Replacing the semipermeable membrane by a dialysis membrane with a cutoff of <20 kDa results in a restriction of the hypoxia-inducing enzymes to the insert system, while oxygen is deployed from the culture medium of the lower compartment containing the cells (Fig. 1G,H). However, it has to be noted that, owing to capillary force, culture medium and enzymes in the upper compartment are dragged towards the protruding end of the dialysis membrane. Therefore, contamination of the lower compartment with enzyme-containing medium from the upper compartment should be avoided by keeping the projecting part of the dialysis membrane as short as possible and/or by bending it inwards.

### Determination of pO_2_

Partial pressure of oxygen (pO_2_) in the culture medium and its temporal decline after the addition of glucose oxidase and catalase was measured by using a flexible probe (LICOX^®^ CMP Oxygen Catheter, Integra, Plainsboro, NJ) that was located in the gap (lower compartment) between the bottom of the six-well plate and the dialysis membrane of the insert (Fig. 1G).

### Glucose measurements

Concentrations of glucose within the culture media were determined using the Fehling’s method (Cresswell, 1886). Briefly, Fehling’s reagents I and II (Sigma, USA) were mixed with the samples and boiled in a water bath for 15 minutes. Absorbance was determined at 495 nm using an ELISA reader (Tecan, Crailsheim, Germany) with Magellan software v1.1. Standard curves were created from known concentrations of glucose.

### Quantification of hydrogen peroxide concentrations

Generation of hydrogen peroxide was quantified with a QuantiChrom^TM^ Peroxide Assay Kit (Bio-Assay Systems, Hayward, CA), which utilizes the chromogenic Fe^3+^-xylenol orange reaction, in which a purple complex is formed when the Fe^2+^ that is provided in the reagent is oxidized to Fe^3+^ by the hydrogen peroxide present in the sample. Briefly, 200 μl of detection reagent were added to 40 μl of sample and measurements were performed based on the manufacturer’s protocol. Samples and controls were measured in a 96-well plate at 620 nm using an ELISA reader (Tecan, Crailsheim, Germany) with Magellan software v1.1. Standard curves were created from known concentrations of hydrogen peroxide.

### Cell culture

Human neuroblastoma cells IMR-32 [LGC Standards, Teddington, UK (Tumilowicz et al., 1970)] were cultured in six-well plates using DMEM/Ham’s F12 with 10% FCS until a confluency of 70–80% was reached. To generate hypoxia, the dialysis membrane devices were filled with glucose-oxidase- and catalase-containing culture medium as described above, and were added to the six wells containing IMR-32 cells. For normoxic controls, enzymes were omitted from the insert. In our previous studies with IMR-32 cells, we employed 2 hours of enzyme-induced hypoxia to yield significant cell damage after 24 hours (Huang et al., 2013; Zitta et al., 2010a). However, in these previous studies, enzymes were added directly to the cell culture medium, resulting in a much faster decrease of oxygen concentrations than in the model described here. Therefore, we decided to use a total of 3 hours hypoxia in the actual experimental setting. After the hypoxic or normoxic period, the inserts were removed, protein was isolated from the cells after 1 and 2 hours, respectively, and LDH measurements were performed after 24 hours (Fig. 3).

### Lactate dehydrogenase (LDH) assays

The colorimetric Cytotoxicity Detection KitPLUS (Roche, Mannheim, Germany) was used for quantifying cytotoxicity by measuring the activity of LDH released from damaged cells. Preparation of samples and measurements were performed based on the manufacturer’s protocol. As in our previous work performed with IMR-32 cells, increased levels of LDH were detected 24 hours after the hypoxic insult (Zitta et al., 2010a); cell culture supernatants were collected at this time point and stored at −20°C. To calculate total LDH, remaining cells were lysed with 2% Triton X-100 (Roth, Karlsruhe, Germany) for 15 minutes to release LDH from the cytoplasm of intact cells. 100 μl of sample and control were evaluated per well of a 96-well plate at 492 nm using an ELISA reader (Tecan, Crailsheim, Germany) with Magellan software v1.1.

### Silver staining

Native culture media, 100× concentrated media as well as recombinant glucose oxidase (5 ng) and catalase (1 μg) were boiled for 5 minutes after addition of sodium dodecyl sulfate–PAGE (SDS-PAGE) sample buffer (62.5 mmol/l Tris-HCl, 2% SDS, 10% glycerol, 5% β-mercaptoethanol; all from Sigma-Aldrich). Concentration of culture media was performed by centrifugation (14,000 ***g***, 30 minutes) using Microcon YM-10 devices (EMD Millipore Corporation, Billerica, MA). Samples were separated by 4–20% gradient (Precise gradient gels, Thermo Scientific, Rockford, IL) SDS-PAGE. Silver staining was performed using the Silver Staining Kit, Protein plus one (GE Healthcare, Munich, Germany), and the protocol provided.

### Western blotting and quantification of protein phosphorylation

After washing the cells with ice-cold PBS, protein extraction was performed using RIPA buffer containing 150 mmol/l sodium chloride, 1.0% NP-40, 0.1% SDS, 1% sodium deoxycholate and 50 mmol/l Tris-HCl (pH 7.6; all from Sigma-Aldrich) with protease and phosphatase inhibitors (Roche). Protein concentrations were determined with a Roti^®^-Quant assay (Roth, Karlsruhe, Germany). Samples were boiled for 5 minutes after addition of SDS-PAGE sample buffer (62.5 mmol/l Tris-HCl, 2% SDS, 10% glycerol and 5% β-mercaptoethanol; all from Sigma-Aldrich). An equal amount of protein (50 μg) of each sample was separated by 10% SDS-PAGE and transferred onto a PVDF membrane (Amersham Pharmacia Biotech, Piscataway, NJ). The membrane was then incubated in TBST buffer (15.4 mM Trizma-HCl, 137 mM NaCl and 0.1% Tween 20) containing 1% BSA (all from Sigma-Aldrich) for 1 hour at room temperature, followed by an overnight incubation with specific antibodies for HIF-1α (# NBP 1-02160; Novus Biologicals, Littleton, CO; 1:3500), procaspase-3 (# sc-7148; Santa Cruz, Heidelberg, Germany; 1:2000), cPARP (# 9542; Cell Signaling, Beverly, MA; 1:1000), catalase (# ab1877; Abcam, Cambridge, UK; 1:15,000), β-actin (# sc-1615; Santa Cruz, Heidelberg, Germany; 1:1000), phospho-Erk1/2 and Erk1/2 (# 9101S and # 9102; Cell Signaling, Boston, MA; 1:8000), phospho-Akt and Akt (# 4060 and # 4619; Cell Signaling, Boston, MA; 1:1000 and 1:2000), phospho-STAT5 and STAT5 (# AF4190 and # AF2168; R&D Systems, Minneapolis, MO; 1:200). After washing in TBST buffer, the membrane was incubated for 1 hour with peroxidase-conjugated swine anti-rabbit (# P0217; Dako, Hamburg, Germany; 1:20,000) or peroxidase-conjugated rabbit anti-goat (# sc-2922; Santa Cruz, Heidelberg, Germany; 1:10,000) immunoglobulin G, referring to the manufacturer’s instructions. The final reaction was visualized using enhanced chemiluminescence (ECL-Plus Western Blotting Detection Reagents; Amersham Pharmacia Biotech, Buckinghamshire, UK), and the membrane was exposed to X-ray films. Images were taken and densitometrically analyzed with the software ImageJ (v1.41o).

### Statistical analyses

All experiments were independently performed 3–5 times. Statistics were done using the statistics software GraphPad Prism version 6.0 for Windows. Data were analyzed by the nonparametric Mann-Whitney test. Variables are expressed as means ± s.e.m.
